# Supplementary material for: Circadian typology is related to emotion regulation, metacognitive beliefs and assertiveness in healthy adults
Source: PLoS One. 2020 Mar 13;15(3):e0230169. doi: 10.1371/journal.pone.0230169 (PMC7069650; doi:10.1371/journal.pone.0230169)
Supplement: Attachment — Submitted filename: Response to Reviewers.docx [file pone.0230169.s001.docx]

Dear Prof Blanch,

Thank you for giving me the opportunity to revise my work for your further consideration. I have considered all the points that have been required by the journal and suggested by the reviewer which has allowed me to improve the manuscript. Next, I reply to each of the comments in a detailed and itemized form.

Reviewer comments:

Reviewer #1:

1. *The author should add a sentence or two (e.g., in the Discussion) asserting to the reader that the differences between evening and morning type are still significant even if age and sex are left out of the model.*

Regarding your suggestion, the assertion that the differences between evening- and morning-type are still significant even if age and sex are left out of the model has been added to the discussion:

“Likewise, it must be noted that differences found between evening and morning-type participants remain significant even once age and sex are removed from the model.”

2. *Minor: Typo “lineal regression” in headings of Tables 4 and 5.*

According to your correction, “lineal regression” has been modified to “linear regression” in both headings of Tables 4 and 5.

Reviewer #2:

1. *“Likewise, significant differences were observed between women (14.78 ± 0.15) and men (13.67 ± 0.11) in rMEQ scores (t(1,2281) = 5.89, p < 0.001, Cohen’s d = 0.25).” Does this mean that more women are morning types?*

Not really, it is needed a score between 18 and 25 in rMEQ to be considered as a morning-type. Nevertheless, it can be said that women are more morning-type than men.

2. *Related to that how does this match with the statements: “Nevertheless, the absence of interactive effect between sex and circadian typology suggests that the circadian typology obtained results are independent of sex.”and “Nevertheless, the non-interaction observed between sex and circadian typology indicates that circadian typology results are independent of sex”. Isn’t the interesting aspect here that morningness-eveningness can contribute to the explanation of emotion regulation, metacognitive beliefs and assertiveness beyond sex as revealed by the regression analysis?*

Absolutely. Both statements are not incompatible with the greater tendency in women toward morningness. Despite this result, no interactive effect between sex and circadian typology in ANCOVA and MANCOVA analyses was found for any of the assessed variables. In other words, although sex exerted an important influence in some variables, carried out analyses revealed that circadian typology was also clearly related to the studied variables regardless of sex, as it has been shown in regression analyses.

3. *The author writes “Participants distribution according to the morningness- eveningness dimension was skewed toward eveningness, which is in line with previous studies performed with large samples [3,4].” Does this mean that we are facing a representative sample here? Further comparisons might be useful. What about the mentioned usual distribution of 20/60/20 % of morning/indifferent/evening types – is this found within the sample?*

The present sample comprised a 27.9% of evening-type, 48.8% of neither-type, and 23.3% of morning-type participants. I assume that the 27.9% rate of evening-type participants might be due to the wide proportion of participants aged 20-30, that differs from the European population (where the wider proportion rounds 35-60 years according to [eurostat](https://ec.europa.eu/eurostat/statistics-explained/index.php/Being_young_in_Europe_today_-_demographic_trends)).

4. *When fatigue is discussed I was wondering whether there is any evidence yet that supports this idea, that evening-types suffer from fatigue?*

Different studies have observed a higher fatigue among evening-type (Fárková, Šmotek, Bendová, Manková, & Kopřivová, 2019; Furusawa et al., 2015; Jeon, Bang, Park, Kim, & Yoon, 2017) . Taking your comment into account, those references have been added to the text. Moreover, as this study did not analyze the fatigue and cause-effect, the fatigue-related phrase has been modified:

“In this line, evening-type persons, who are the mainly social jet-lag sufferers, must perform different functions (sleep and wake-up, eat, work, study, child care, etc.) in a schedule that is unadjusted to their biological clock, which might be understood as upstream swim, that might result in higher fatigue as it has been observed in previous works [83–85].”

5. *If I got the reasoning right that is deduced from social jet-lag theory evening-types (working against their biological clock) that have to adapt to a more morning-oriented social clock should experience greater fatigue which brings up maladaptive cognitive, emotional and behavioral habits. What about other groups of persons or cultures?*

It will always depends on the synchrony between the social (cultural) clock and the biological clock of any individual.

6. *I think the english language still needs some corrections e.g. “…non-found…”; “…the hypothesis that evening-type participants show(s)…”; “One of them is the lower absence of control of the on-line data collection…*

Attending to your comment, corrections have been performed.

7. *I was wondering how many participants were excluded due to duplicate answers and how many because they did not meet the inclusion criteria.*

Fifty-one duplicate answers were found. First, data was introduced into a Microsoft Excel sheet and a function that highlights all duplicate rows was performed. Second, the absence of very similar responses was checked manually. For this purpose, I checked responses that were sent almost simultaneously. Finally, email duplicates were looked for. For those, the similarity of the replies to the different questionnaires, specially to sociodemographic data, was checked. In the event that sociodemographic data were different, this was not considered as a duplicate answer.

8. *Also the inclusion/exclusion criteria should be named half a page earlier when the exclusion is mentioned.*

According to your recommendation, inclusion/exclusion criteria has been named following the exclusion mention:

“The total amount of participants in the study was 3016, out of which 733 were excluded due to duplicate answers and for non-accomplishing the inclusion criteria (Spanish resident, absence of mental and physical health problems, absence of shift work and 18-60 yrs).”

9. *Does inclusion/exclusion significantly change the results of the present study?*

Yes. If exclusion criteria are not applied the obtained results are stronger in terms of new significant associations as well as in term of statistical results (*p*, R^2^, F, partial eta square and observed power).

10. *Please explain the score range for the rMEQ. What are the scales?*

rMEQ is a 25-items questionnaire that can be scored between 4 and 25. The lower the scores are, the closer one is to the eveningness pole; The higher the scores are, the closer one is to the morningness pole. Moreover, this questionnaire allows to classify participants into three circadian typology categories: evening-type (for scores between 4 and 11), neither-type (for scores between 12 and 17), and morning-type (for scores between 18 and 25).

11. *Decimal separators are inkonsistent in the participants section and in Table 1.*

Attending to your comment, decimals separators in participants section and in Table 1 have been corrected.

12. *“…and evening-type (27.49 ± 0.30 yrs; p < 0.001), who were older than neither-type (p < 0.001).” I believe it should read younger, if the the numbers are in brackets are means.*

Attending to your correction, older has been changed to younger.

13. *The metacognitions sections would be easier to follow when all post-hoc comparisons would follow the description of significance directly.*

Attending to your recommendation, metacognitions section has been modified as follows:

“Significant main effects were observed in MCQ-30 total score, negative beliefs of uncontrollability and danger, cognitive confidence and cognitive self-consciousness for circadian typology. According to post-hoc comparisons, neither-type participants showed higher scores than morning-type in cognitive confidence (MD = 0.70, *p* = 0.012, Cohen’s *d* = 0.12) and scored lower than evening-type in MCQ-30 total score (MD = 1.94, *p* = 0.032, Cohen’s *d* = 0.21) and cognitive self-consciousness (MD = 0.65, *p* = 0.016, Cohen’s *d* = 0.23). Evening-type subjects showed higher scores than morning-type in MCQ-30 total score (MD = 3.13, *p* = 0*.*002, Cohen’s d = 0.17), negative beliefs of uncontrollability and danger (MD = 0.91, *p* = 0*.*002, Cohen’s d = 0.23), cognitive confidence (MD = 0.97, *p* = 0*.*002, Cohen’s d = 0.17) and cognitive self-consciousness (MD = 0.74, *p* = 0*.*02, Cohen’s d = 0.39).Regarding sex, significant main effects were observed in positive beliefs about worry, need to control thoughts and cognitive self-consciousness. In this line, men scored higher than women in positive beliefs about worry (MD = 1.27, *p* > .001, Cohen’s d = 0.35), need to control thoughts (MD = 1.29, *p* > .001, Cohen’s d = 0.38) and cognitive self-consciousness (MD = 0.74, *p* > .001, Cohen’s d = 0.23).”

14. *I think the section regression analysis is not easy to follow linguistically as links are not clear.*

Attending to your recommendation, regression section has been modified as follows:

“Table 3 shows results of multiple regression analyses while Tables 4 and 5 show the model coefficients for each variable. The analyses revealed that sex and age were significantly related to cognitive reappraisal (explaining a 0.4% of the variance), expressive suppression (14.2%), assertiveness (0.7%), metacognitions (3.9%), positive beliefs about worry (5.2%), need to control thoughts (5.5%) and cognitive self-consciousness (6.6%), with *p* < .05 in all cases. Moreover, the inclusion of the rMEQ scores in the equation significantly increased the explained variance for cognitive reappraisal (0.5%), expressive suppression (0.3%), assertiveness (0.6%), metacognitions (0.8%), negative beliefs of uncontrollability and danger (0.9%), cognitive confidence (0.7%), need to control thoughts (0.3%) and cognitive self-consciousness (0.5%), with *p* < .05 in all cases.”

15. *Also: was the increase from the first to the second model significant?*

Significant increases from the first to the second model was observed for cognitive reappraisal, expressive suppression, assertiveness, metacognitions, negative beliefs of uncontrollability and danger, cognitive confidence, need to control thoughts and cognitive self-consciousness. Regarding your comment, the fact that the inclusion of the rMEQ scores in the equation resulted in a significantly increase of the explained variance was added to this section:

“Moreover, the inclusion of the rMEQ scores in the equation resulted in a significantly increase of the explained variance for cognitive reappraisal…”

16. *Table 2 should be ordered the same way as the results section.*

According to your recommendation, Table 2 has been ordered the same way as the results section

17. *Table 4 und 5 are named alike. Aren’t the information in Table 5 subscales of metacognitive beliefs?*

Attending to your comment, tables 4 and 5 have been renamed as follows:

“Table 4. Multiple linear regression model coefficients for emotion regulation strategies, assertiveness and MCQ-30 total score.”

“Table 5. Multiple linear regression model coefficients for the different metacognitions assessed through MCQ-30.”

18. *Please provide english explanations for the variables in the .sav-file on OSF.*

The file with English explanations for the variables can be found at <https://osf.io/nh5c4>
